# Synthesis and Characterization of Fe-TiO_2_ Nanomaterial: Performance Evaluation for RB5 Decolorization and In Vitro Antibacterial Studies

**DOI:** 10.3390/nano11020436

**Published:** 2021-02-09

**Authors:** Muhammad Saqib Khan, Jehanzeb Ali Shah, Nadia Riaz, Tayyab Ashfaq Butt, Asim Jahangir Khan, Walid Khalifa, Hatem Hassin Gasmi, Enamur Rahim Latifee, Muhammad Arshad, Ahmed Abdullah Alawi Al-Naghi, Anwar Ul-Hamid, Muhammad Arshad, Muhammad Bilal

**Affiliations:** 1 Department of Environmental Sciences, Abbottabad Campus, COMSATS University Islamabad, Abbottabad 22060, Pakistan; muhammadsaqib@yahoo.com (M.S.K.); jehanzeb360@yahoo.com (J.A.S.); nadiariazz@gmail.com (N.R.), asimjkw@gmail.com (A.J.K.); 2 Department of Civil Engineering, University of Hail, Hail 55476, Saudi Arabia; ta.butt@uoh.edu.sa (T.A.B.); w.khalifa@uoh.edu.sa (W.K.); h.gasmi@uoh.edu.sa (H.H.G.); E.Latifee@uoh.edu.sa (E.R.L.); a.alnaghi@uoh.edu.sa (A.A.A.A.-N.); 3 National Center for Physics, Nanosciences and Technology Department, Quaid-i-Azam University Islamabad Campus, Islamabad 44000, Pakistan; Arshad_pr2002@yahoo.com; 4 Centre for Engineering Research, King Fahd University of Petroleum and Minerals, Dhahran 31261, Saudi Arabia; anwar@kfupm.edu.sa; 5 Institute of Environmental Sciences and Engineering, School of Civil and Environmental Engineering, National University of Sciences and Technology, Islamabad 44000, Pakistan

**Keywords:** water disinfection, photo inhibition activity of TiO_2_, RB5 reaction kinetics

## Abstract

A photocatalytic system for decolorization of double azo reactive black 5 (RB5) dye and water disinfection of *E. coli* was developed. Sol gel method was employed for the synthesis of Fe-TiO_2_ photocatalysts and were characterized using thermogravimetric analysis (TGA), Fourier transform infrared spectroscopy (FTIR), X-ray diffraction (XRD), scanning electron microscopy (SEM) coupled with energy dispersive X-ray analysis (EDX), transmission electron microscopy (TEM), diffuse reflectance spectroscopy (DRS) and Brunauer–Emmett–Teller (BET) analysis. Results showed that photocatalytic efficiency was greatly influenced by 0.1 weight percent iron loading and 300 °C calcination temperature. The optimized reaction parameters were found to be the ambient temperature, working solution pH 6.2 and 1 mg g^−1^ dose to completely decolorize RB5. The isotherm studies showed that RB5 adsorption by Fe-TiO_2_ followed the Langmuir isotherm with maximum adsorption capacity of 42.7 mg g^−1^ and K_ads_ 0.0079 L mg^−1^. Under illumination, the modified photocatalytic material had higher decolorization efficiency as compared to unmodified photocatalyst. Kinetic studies of the modified material under visible light irradiation indicated the reaction followed the pseudo-first-order kinetics. The illumination reaction followed the Langmuir-Hinshelwood (L-H) model as the rate of dye decolorization increased with an incremental increase in dye concentration. The L-H constant K_c_ was 1.5542 mg L^–1^∙h^–1^ while K_ads_ was found 0.1317 L mg^–1^. The best photocatalyst showed prominent percent reduction of *E. coli* in 120 min. Finally, 0.1Fe-TiO_2_-300 could be an efficient photocatalyst and can provide a composite solution for RB5 decolorization and bacterial strain inhibition.

## 1. Introduction

Wastewater generation whether industrial or domestic by rapid industrialization and/or urbanization and its discharge into natural drainage system has severely affected the fragile aquatic environment and thus becoming the principal source of toxic contaminants and pathogen dissemination. Higher volume of wastewater loaded with complex and versatile nature of contaminants including dyes is hot environmental issue and aggravating the environmental concerns around the globe. Strict effluent discharge laws have made the industry and wastewater researchers to explore the efficient technologies for the provision of composite treatment solutions which could meet simultaneously the dye decolorization and pathogens killing and ultimately meet the safe water quality standards. Currently different conventional methods are employed for industrial wastewater treatment including, biological oxidation and physico-chemical methods, coagulation/flocculation [1], reverse osmosis [2], membrane filtration [3], activated carbon adsorption [4,5]. All the above methods are pollutant specific and are not capable to deactivate the harmful pathogen, more precisely, biological method provide favorable conditions for these harmful human pathogens. Moreover, the non-destructive action nature and just transferring the contamination from one phase to another as well as secondary waste generation and further necessity of treatment or pretreatment process disfavor the physico-chemical processes adoption [6,7].

Advanced oxidation process (AOP) is best way to fully decolorize organic pollutants and deactivate harmful pathogens as the non-selective nature of OH radicals offer this approach an additional benefit. The heterogenous photocatalytic system follows the AOP at the surface of photocatalysts due to production of electrons (e^−^) and holes (h^+^) in the conduction and valence bands through excitation of photons and this charge separation contributes to the production of OH radicals. TiO_2_ based photocatalysts are considered best heterogenous photocatalysts due to the non-toxicity and availability. However, the only hinderance in large scale application is activation requirement in UV region of the spectrum. To overcome this problem, researchers have done marvelous work in reducing bandgap through doping with impurities like Fe, Ni, Cu, N, P and S [8,9,10].

Doping with iron(III) has been widely investigated among different metal ions because of its distinctive electronic structure and size, that closely matches the titanium (IV) [11,12,13]. The electronic states of iron ions in titania lead to the creation of effective electron and holes trapping sites leading to the enhanced photocatalytic activity [14,15]. However, the effect of metal doping on photocatalytic activity of the synthesized nanomaterial depends on various factors including synthesis method, calcination temperature and doping level [16,17]. Different synthesis procedures have been adopted for synthesis of iron doped TiO_2_, including hydrothermal [18], solvothermal, wet impregnation [19], co-precipitation [20,21] and sol gel method [22,23]. Sol gel method is regarded as the most employed popular method to control the particle size and crystallinity [21]. Table 1 compares different Fe doped TiO_2_ studies reported previously.

Although a lot of research work has been reported for air and water purification through TiO_2_ photocatalysts, but a little attention has been given to water decontamination and remediation of various kinds of microbial contaminants using these semiconductors. Recently, some researcher considered Ag doped metal oxides for destroying human pathogens due to the antimicrobial activity of Ag [30,31] but the other doped metal or nonmetal has not yet been explored for their antimicrobial activity. Current investigation focused the synthesis and characterization of Fe-TiO_2_ photocatalyst and its application in decolorizing the double azo reactive black 5 (RB5) dye, but focus has been given to explore the potential of Fe doped TiO_2_ for deactivation of model human pathogen *E. coli*. Initially Fe-TiO_2_ was screened out for Fe loading and calcination temperatures and the best combination was optimized for RB5 decolorization and *E. coli*.

## 2. Materials and Methods

### 2.1. Materials

Titanium tetra-isopropoxide (TTIP) with a purity of 98% was supplied by Daejung, South Korea. Absolute ethanol and glacial acetic acid with a purity of 99% were purchased from Merck Darmstadt, Germany. Deionized water was produced using B114 deionizer in the laboratory. Iron nitrate and the commercial reactive black 5 (RB5), an azo dye, were acquired from Sigma Aldrich, Munich, Germany.

### 2.2. Synthesis of TiO_2_ and Fe-TiO_2_ Photocatalysts

The modified sol-gel method was used to synthesize TiO_2_ photocatalysts [32]. Precisely, 37 mL TTIP was poured to 60 mL absolute ethanol and designated as solution A. In addition, a second solution B was generated by blending 10 mL deionized water and 15 mL acetic acid in 20 mL absolute ethanol. Under intense stirring, solution B was added dropwise to solution A. The solution was stirred at room temperature (25 °C ± 1) until gel was formed. The obtained gel was aged for 24 h under ambient conditions, dried in oven (UN 30, Memmert-Kupfer, Dominik, Germany), and ground to powder. Fe-TiO_2_ photocatalysts were synthesized by the modified synthesis method. The iron precursor was introduced to solution B prior to adding solution B to solution A, pursued by the process as reported above. Different Fe weight percent including 0.01, 0.05, 0.1, 0.5, 1 and 5 were synthesized. The photocatalysts sample were denoted as mFe-TiO_2_-T, where small m represents the weight percent, Fe represents the iron, TiO_2_ shows the titanium and capital T represents the calcination temperature for example 0.1Fe-TiO_2_-200 shows the 0.1 weight percent iron loading onto titania and calcined at 200 °C.

### 2.3. Photocatalyst Characterization

The best performing photocatalysts were chosen for characterization of the various physicochemical properties like thermal stability, functional groups, identification of phases and crystallite size, surface morphology, bandgap estimation and surface area analysis using thermal gravimetric analyses (TGA-STA 8000, Boston, Massachusetts, United States), Fourier-transformed infrared spectroscopy (FTIR-Alpha Bruker, Karlsruhe, Germany), X-ray diffraction (XRD-Bruker, Billerica, Massachusetts, United States), scanning electron microscopy coupled with energy dispersive X-ray analysis (JEOL, JSM-6510LA, Tokyo, Japan), transmission electron microscopy (TEM), and diffuse reflectance spectroscopy (DRS-UV-2600i, Kyoto, Japan) and Brunauer–Emmett–Teller (BET) analyses, respectively. XRD was realized at 40 kV, 40 mA in the scanning angle (2θ) range of 10–80° at scan rate of 2° min^−1^ using diffractometer equipped with a Cu K_α_ radiation source. The standard diffraction data was compared, and unknown components were recognized. Scherrer formula (Equation 1) was utilized for estimation of particle sizes (D) of nanomaterial [33].

(1) $$D=\frac{K\lambda }{\beta cos\theta }$$

The Scherrer constant (*K*) represents the particle shape and usually the *K* value is considered to be 0.9 [34], *λ* exhibits the wavelength, *θ* indicates the diffraction angle and *β* denotes the full width at half maximum (FWHM) of the reflection peak.

### 2.4. RB5 Decolorization

RB5 decolorization was investigated at 30 ppm of dye, initial pH 6.2 and Fe-TiO_2_ dose of 1 g L^−1^ under a visible light source at ambient temperature. The required amount of RB5 was taken to constitute 30 ppm solution in total volume of 30 mL. Initially the Fe-TiO_2_ was weighed and blended with distilled water followed by 10 min of ultrasonication. For the dark reaction, the mixture was stirred with a magnetic stirrer for 30 min, and later the same suspension was illuminated for 60 min under visible light source of 500 W with 30798 lux light intensity (Halogen lamp, Hi Luminar-Germany,) at 25 cm distance. Figure S1, supplementary information represents the light spectrum reported in current investigation. RB5 adsorption (dark) and decolorization (light) was monitored in the samples collected at pre-determined time intervals. 

### 2.5. Optimization Studies

RB5 decolorization was examined through absorbance measurements at 598 nm wavelength by UV-visible spectrophotometer (PG instruments T80^+^, Lutterworth, UK). The standard solutions of RB5 with 1, 10, 20, 30, 50, 60 and 100 ppm concentrations were used to develop the calibration curve. The reaction mixture was centrifuged to remove suspended particles of photocatalysts each time prior to the absorbance measurement. RB5 decolorization efficiency was determined using the Equation (2).

(2) $$RB5\;Decolorization\left(\%\right)=\left(\frac{C_{0}-C_{t}}{C_{0}}\right)100$$

where C_0_ and C_t_ indicate, respectively, the initial and the residual RB5 concentration at time, *t*. The photocatalytic system was optimized based on RB5 decolorization investigations for reaction parameters including the contact time, pH, Fe-TiO_2_ dose and RB5 concentration.

### 2.6. Adsorption Isotherms

The best performing photocatalyst, 0.1Fe-TiO_2_-300, were used to study the adsorption pathways of RB5 in the dark. The two well-known adsorption isotherms namely Freundlich and Langmuir were fitted to RB5 adsorption data analyzed under in the dark and the mechanism of RB5 adsorption was delineated. The linearly transformed Langmuir model (Equation (3)) was applied to determine the value of *Q_m_* and *K_ads_* from the intercept (*1/Q_m_*) and slop (*1/Q_m_·K_ads_*) of plot 1/*Q_e_* versus1/*C_e_*.

(3) $$\frac{1}{Q_{e}}=\frac{1}{Q_{m}}+\left(\frac{1}{K_{ads}Q_{m}}\right)\frac{1}{C_{e}}$$

The linear expression of Freundlich model can be represented by Equation (4).

(4) $$\ln\left(Q_{e}\right)=\ln\left(K_{F}\right)+\frac{1}{n}\ln C_{e}$$

*Q_e_* (mg·g^−1^) indicates the quantity of RB5 adsorbed per unit weight of Fe-TiO_2_ at the equilibrium time, *Q_m_* (mg·g^−1^) exhibits the maximum adsorption capacity of Fe-TiO_2_ for RB5, *C_e_* (mg·L^−1^) denotes the residual concentration of the dye at equilibrium. *K_ads_* (L·mg^−1^) indicates Langmuir adsorption constant. The Freundlich constants, i.e., *K_F_* and n, exhibit the adsorption capacity and heterogeneity factor, respectively.

### 2.7. Photocatalytic Kinetics

In the presence of Fe-TiO_2_ photocatalyst, the Langmuir-Hinshelwood model [35] can be employed to elaborate the rate of the photocatalytic decolorization of RB5 dye over time. Langmuir-Hinshelwood model for photocatalytic system can be explained as:

(5) $$\frac{1}{r_{0}}=\frac{1}{k_{c}}+\frac{1}{k_{c}K_{LH}}.\frac{1}{{\left[RB5\right]}_{e}}$$

The dependency of 1/r0 for the corresponding *1/[RB5]_e_* concentration values of RB5 can be translated by Equation (5). In comparison, the k_c_ and K_LH_ values demonstrate the effect of the RB5 concentration on the equilibrium constant. 

### 2.8. Photocatalytic Disinfection Performance Evaluation

To check the photocatalytic disinfection ability of the Fe-TiO_2_ photocatalyst, bactericidal activity was conducted using the best performing Fe-TiO_2_ photocatalyst (screened from RB5 decolorization experiments). Antibacterial activities of Fe-TiO_2_ photocatalyst were tested using different parameters (irradiation time and photocatalyst calcination temperatures) against *Escherichia coli* (ATCC-25922) as model pathogen. Detailed antimicrobial protocol followed is reported in our recent publication [30,36]. Data were presented with the following formula in relation to a percent reduction before and after inhibition treatment of model pathogen.

(6) $$Reduction\;\left(\%\right)\;=\;\left(\frac{A-B}{A}\right)\times 100$$

A and B reveal the number of viable bacteria, respectively, before and after photocatalytic oxidation. 

### 2.9. Energy Efficiency and Cost Analysis

Energy efficiency was estimated through electrical energy consumption (EE/O) for the best synthesized photocatalysts from each combination using the following equation [37].

(7) $$E_{E/O}=\frac{\left(pt\right)1000}{[\left(V\right)60\ln\left(\frac{C_{0}}{C_{f}}\right)}$$

## 3. Result and Discussion

### 3.1. Thermal Analysis

**Figure 1:**
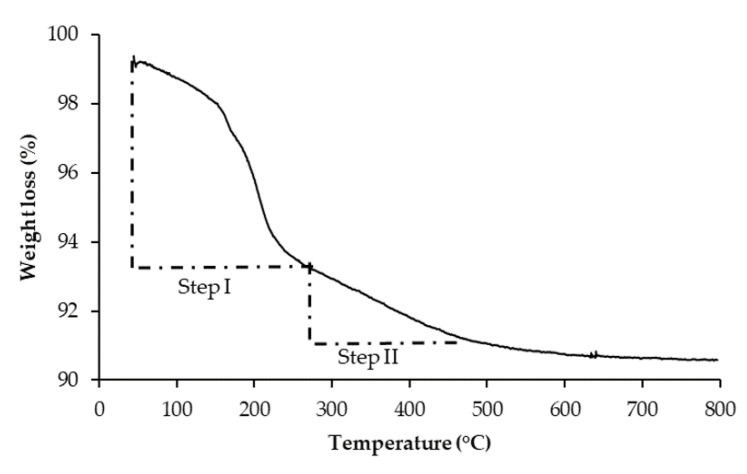
TG weight loss profile of 0.1Fe-TiO_2_ photocatalyst.

Thermogravimetric studies were conducted for as synthesized raw 0.1Fe-TiO_2_ photocatalyst to select the suitable calcination temperature. Figure 1 shows the weight loss profile of raw 0.1Fe-TiO_2_ photocatalyst. Two weight loss steps are evident from the TGA profile, 5.98% weight loss occurred in step I that is from room temperature to 260 °C while 2.30% weight loss was observed in step II from 260 to 490 °C. Total weight loss in these two steps was 8.28%.

In step I, from room temperature to 200 °C, solvents were evaporated while thermal decolorization of loosely bound organics occurred up till 260 °C. In step II, thermal decomposition of tightly bound organic residues and crystallization of the anatase phase occurred above 260 °C. Our results support the previous work [38].

### 3.2. Functional Group Identification

**Figure 2:**
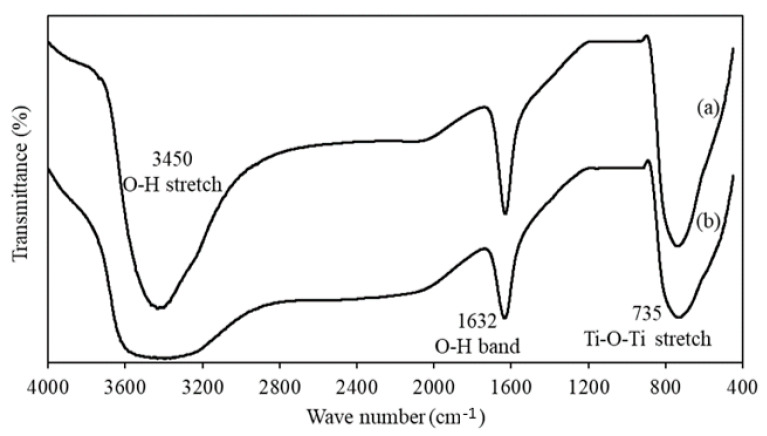
FTIR spectra of (**a**) TiO_2_-300; (**b**) 0.1Fe-TiO_2_-300.

The FTIR spectra provides evidence about the molecular geometry and interactions of the functional groups existing in the system. FTIR spectra of TiO_2_-300 and 0.1Fe-TiO_2_-300 photocatalysts can be observed in Figure 2. The band at 3450 cm^−1^ is ascribed to the stretching mode of OH group on the TiO_2_ in both samples [39], corresponds to the presence of water molecules. The OH group serves as a scavenger for the produced charge carrier, leading OH radical formation. In decolorization of the RB5 dye, this OH radical plays a major role as they are highly reactive species with high redox potential (2.8 V), and able to oxidize soluble inorganic and organic substances. FTIR spectrum also indicates that the absorbance rate is higher in the IR region (1632 cm^−1^) [36], indicating the surface hydroxylation upon doping TiO_2_ with Fe. Ti-O stretching appeared between 520–735 cm^−1^ [40]. These bands serve an active role in improving the efficiency of photocatalysts for RB5 decolorization [39].

### 3.3. X-ray Diffraction Analysis

**Figure 3:**
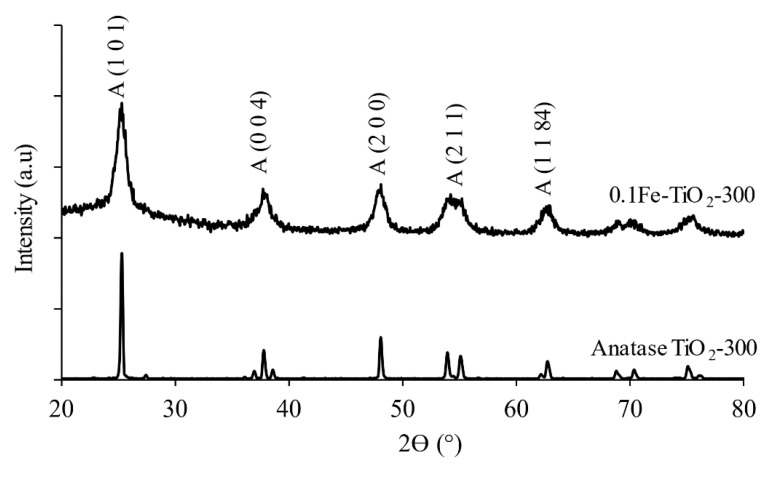
XRD peaks of anatase TiO_2_-300 and 0.1Fe-TiO_2_-300.

Figure 3 displays X-ray diffraction analysis of TiO_2_ (pure anatase) and 0.1Fe-TiO_2_-300 nanomaterials. The diffraction peaks are well allocated to the crystalline TiO_2_ anatase (JCPDS 84-1286). In addition, no peaks for the rutile and brookite phases were observed at 0.1Fe-TiO_2_-300. The development of TiO_2_ crystals is evident in the anatase phase of TiO_2_ as the XRD peak intensities are weakened and expanded upon doping with iron. Similar observations were documented for Fe-N/TiO_2_ [41]. No indications were seen for the presence of Fe species in all the XRD patterns. This may be due to high dispersion of Fe species and the low metal content. This is well explained in our previous studies [33,42]. The FWHM of prominent anatase (2θ = 25.2°) exhibits the (1 0 1) plane diffraction. The crystallite size was calculated using equation (1). The broad peaks confirm the occurrence of small crystallite having mean size of 5.93 nm and 45.11 nm, respectively, for 0.1Fe-TiO_2_-300 and TiO_2_ anatase. Crystallite size for 0.1Fe-TiO_2_-350 was exhibited as 9.91 and reduction in crystallite size of TiO_2_ by Fe doping has also been reported previously [43,44].

### 3.4. Scanning Electron Microscopy (SEM) and Transmission Electron Microscopy (TEM) Analyses

**Figure 4:**
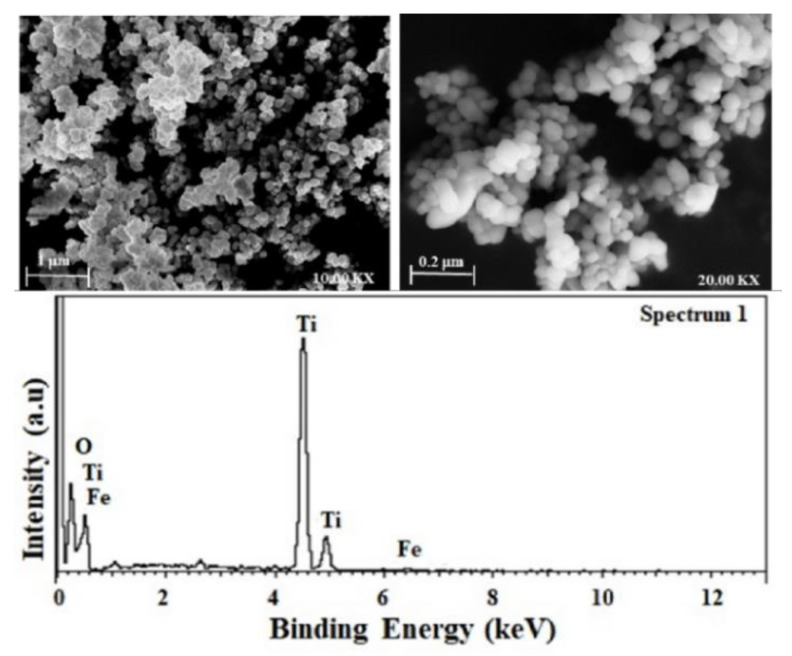
Scanning electron micrographs (SEM-EDX) of 0.1Fe-TiO_2_ calcined at 300 °C.

SEM was used to study the crystallite shape, size, and metal dispersion while elemental composition was quantified using EDX analysis. Figure 4 demonstrates the spherical morphology of particles with increased agglomeration, and no localized metal particles have been observed exhibiting high iron dispersion on the surface of TiO_2_. The EDX spectrum shows very small intensities of Fe in the 0.1Fe-TiO_2_-300 photocatalysts. Similar observations were reported previously for different Fe doped TiO_2_ photocatalysts [38,45]. 

**Figure 5:**
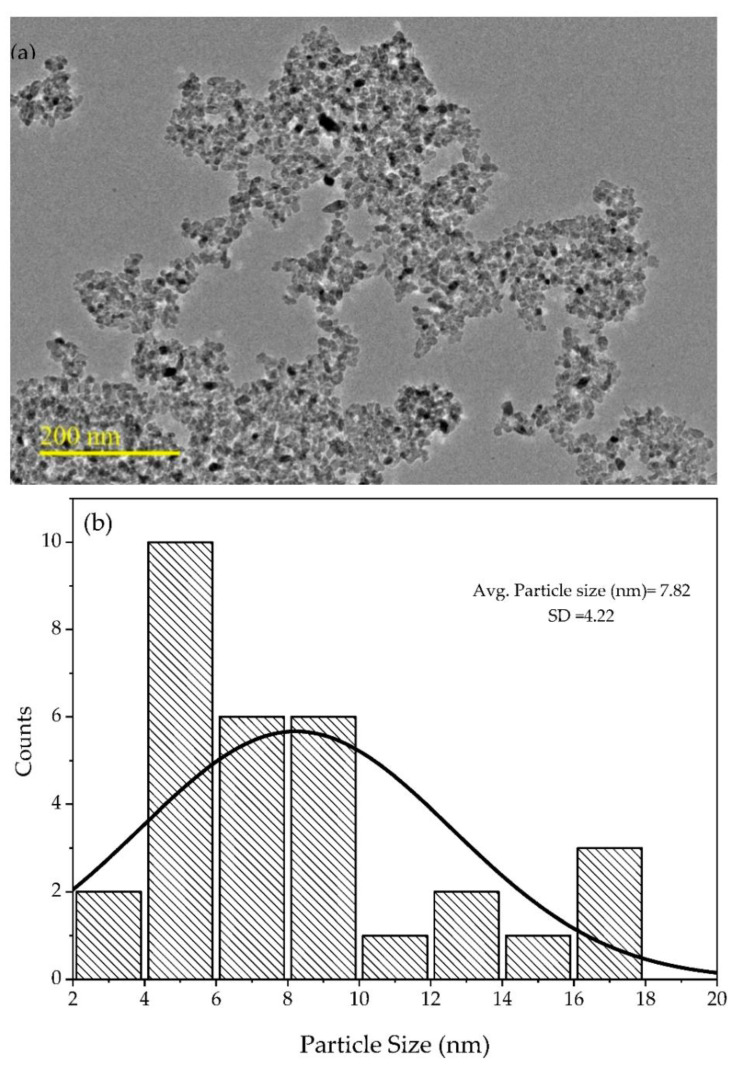
(**a**)TEM micrographs and (**b**) size distribution histogram of 0.1Fe-TiO_2_-300.

TEM micrograms have been used to observe the microstructure for further study of individual grain and grain boundaries. TEM micrographs of 0.1Fe-TiO_2_-300 photocatalysts are displayed in Figure 5a while histogram of particle size distribution is shown in Figure 5b. It can be seen from the TEM micrographs that particles adhere to each other and are in good agreement with SEM images. The average particle size of 0.1Fe-TiO_2_-300 was 7.82 ± 4.22 nm. These results are in close agreement with that obtained for crystallite size in XRD analysis. The particles stickiness can be associated to different phenomenon, as explained by previous study [46]. Moreover, in one of the previous works by Solani et. al., 2019, the average crystalline size reported was 13 ± 2.52 nm [44].

### 3.5. Bandgap Analysis

**Figure 6:**
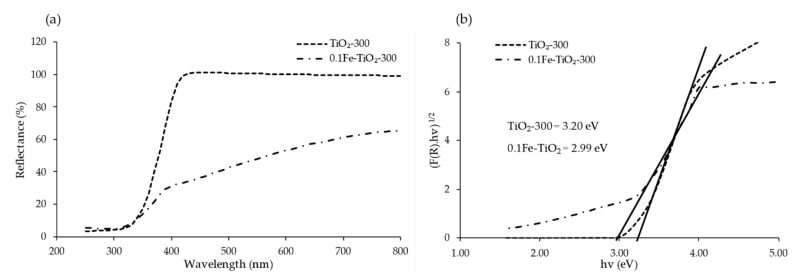
(**a**) Reflectance spectrum; (**b**) Tauc plot for bandgap estimation of TiO_2_-300 and 0.1Fe-TiO_2_-300 photocatalysts.

The task of allowing TiO_2_ to operate in the visible light source is to modify its lattice structure by adding some impurities. Diffuse reflectance spectroscopy was used to observe this shift. Figure 6a shows the reflectance spectrum of TiO_2_-300, and 0.1Fe-TiO_2_-300 photocatalysts. A visible shift of the optical absorption thresholds was observed for 0.1Fe-TiO_2_-300 compared to TiO_2_-300. The sharp absorption edge of around 390 nm was assigned to the excitation of the electron from VB to CB [47]. Tauc model is generally used to describe the light absorption process of amorphous semiconductors and estimate the band gap [48]. From the plot of (F(R).hv)^1/2^ versus hv, the photocatalysts’ bandgap energy was calculated. The photocatalyst’s bandgap yielded by extrapolating it to the tangent of the graph in the low energy range (hv) axis when [F(R).hv]^1/2^ = 0 as shown in Figure 6b, while method of estimation is explained in Figure S2. The bandgap TiO_2_-300, and 0.1Fe-TiO_2_-300 photocatalysts was found to be 3.20 and 2.99 eV, respectively. This demonstrates a significant increase in the light absorption ability in the visible region for the iron-doped TiO_2_ sample.

### 3.6. BET Analysis

**Figure 7:**
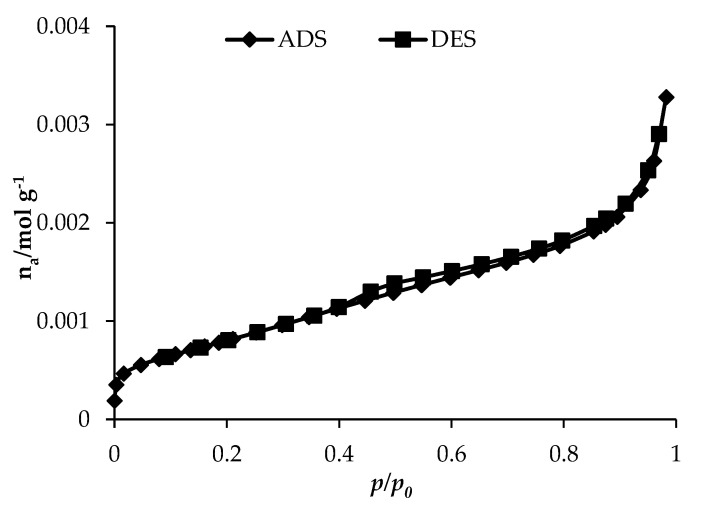
N_2_ adsorption/desorption isotherms of the 0.1Fe-TiO_2_-300 photocatalyst.

Figure 7 demonstrate the nitrogen adsorption-desorption curve for 0.1Fe-TiO_2_-300. The adsorption isotherms of nitrogen at 77 K were obtained using eight values of relative pressure ranging from 0.05 to 1. Pore size distribution curve was calculated from the desorption (DES) branch of the nitrogen isotherm by the Barrett–Joyner–Halenda (BJH) method and the corresponding nitrogen adsorption–desorption isotherms (ADS-DES) of the photocatalysts [49,50]. BET type II curve was observed, indicating the mesoporous nature of 0.1Fe-TiO_2_-300 photocatalyst with mean pore diameter of 6.83 nm. In type II isotherm the flat region in the middle represents the formation of monolayer. Such mesoporous photocatalysts are preferred generally for photocatalytic decolorization because higher porosity of photocatalysts favors adsorption of dye molecules. BET results showed the surface area for 0.1Fe-TiO_2_-300 as 70 m^2^ g^−1^ with pore volume of 0.115 cm^3^ g^−1^. Results from adsorption studies best fit with Langmuir adsorption isotherm showing the formation of monolayer chemisorption mechanism, which agree with N_2_ adsorption desorption analysis.

### 3.7. Photodecolorization Studies

#### 3.7.1. Effect of Photocatalyst Dose

**Figure 8:**
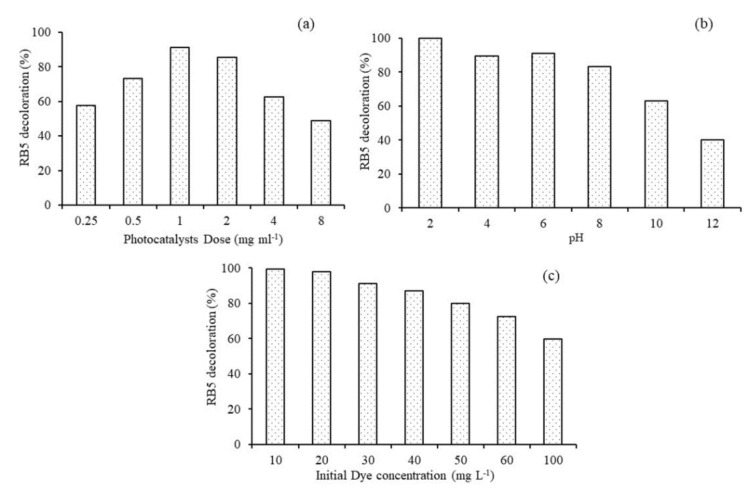
Effect of (**a**) photocatalyst dose; (**b**) pH; (**c**) initial dye concentration on the percent decolorization of RB5 azo dye using 0.1Fe-TiO_2_-300 °C.

Mass of the photocatalyst is directly proportional to the rate of reaction, however after a certain amount of dose the reaction rate levels off. This phenomenon is well explained in previous studies through masking of the photocatalyst surface by high dose [19,51,52]. Therefore, it is essential to optimize the photocatalytic system with optimum dose of the photocatalysts. Experiments were performed with different 0.1Fe-TiO_2_-300 dose i.e., 0.25, 0.5, 1, 2, 4 and 8 mg mL^−1^. Figure 8a shows that there is an increase in % RB5 decolorization from 57% to 91% with an increase in 0.1Fe-TiO_2_-300 dose from 0.25 mg mL^−1^ to 1 mg mL^−1^ but after 1 mg mL^−1^ decrease in % RB5 decolorization was observed and at 8 mg mL^−1^ only 48% RB5 decolorization was exhibited. It was also observed during the experimental proceedings that at higher dose of 0.1Fe-TiO_2_-300 the solution become turbid and light penetration into the solution was hindered, so lower e^−^/h^+^ generation can be assumed, which resulted in lower % RB5 decolorization. Moreover, previous studies reported the aggregation of nanoparticles at higher dose thus reduces the photocatalytic activity [53,54]. Therefore, the optimum dose of 0.1Fe-TiO_2_-300 was chosen as 1 mg mL^−1^.

#### 3.7.2. Effect of Reaction pH

The photocatalyst’s surface charge, the acid base property of the metal oxide and the generation and scavenging of hydroxyl radicals are affected due to the amphoteric behavior of the semiconductor, eventually affecting the semiconductor’s decolorization efficiency. Hence it is important to optimize the photocatalytic performance of 0.1Fe-TiO_2_-300 under different pH conditions. Figure 8b depicts the effect of variable pH and optimum dose of 1 mg mL^−1^ on decolorization of 0.1Fe-TiO_2_-300 photocatalyst for RB5 dye. Best results were obtained for lower pH as compared to higher pH. 100% decolorization was observed at pH 2 while 40% decolorization of RB5 dye was observed at pH 12. The effect of pH can be explained based on point of zero charge (PZC) on the surface of using 0.1Fe-TiO_2_-300 under different pH conditions. The PZC for Fe-TiO_2_ is between 5.6 and 6.4 [55], the Fe doped photocatalyst shall be positively charged in acidic medium while it shall be negatively charged in alkaline medium. The higher decolorization of anionic RB5 dye under acidic and lower under alkaline conditions are best explained by the above phenomenon. Due to similar charges on the surface of the photocatalysts and the pollutant, the electrostatic repulsion between similar charges reduces the efficiency of the system as explained in previous studies [51,56,57]. A similar study on rhodamine B photocatalysis by Fe-TiO_2_ reported that the performance of the photocatalyst was increased when initial pH was augmented from 2.0 to 6.0, because the charge of the catalyst surface was opposite to that of rhodamine B and, thus, the attraction tendency was observed high [56,58].

#### 3.7.3. Effect of Initial Dye Concentration

It is essential to investigate the effect of pollutant concentration on the photocatalyst’s decolorization efficiency from an application point of view. It is widely accepted that with an increase in dye concentration to a certain level, photocatalytic decolorization increases and further increase lower the decolorization efficiency of photocatalytic. Figure 8c illustrates the effect of different initial RB5 concentration at the working pH and 1 mg mL^−1^ of photocatalyst dose on percent RB5 decolorization. Initially at lower concentration, 10 and 20 and 30 mg L^−1^, the decolorization was observed to be 100 and 91% but further increase in RB5 concentration, 40, 50, 60 and 100 mg L^−1^, the percent decolorization decreased from 87%, 80%, 72% and 60%, respectively. The reduction in photocatalytic efficiency of 0.1Fe-TiO_2_-300 with increase in RB5 concentration can be attributed to different reasons as stated in previous studies like non-availability of active adsorption sites due to high pollutant load, interference in light penetration to the surface of 0.1Fe-TiO_2_-300 photocatalysts for activation and lower radical production to proceed the photocatalytic process [51,56,59,60]. Shima et al. reported the possible reason that when the initial concentration is increased, more dye molecules are adsorbed on the surface of the Fe doped TiO_2_, thus all the surface sites for the adsorption of hydroxyl ions are blocked and, hence, lower tendency of the generation of hydroxyl radicals [57].

#### 3.7.4. Fe-TiO_2_ Adsorption Studies

**Figure 9:**
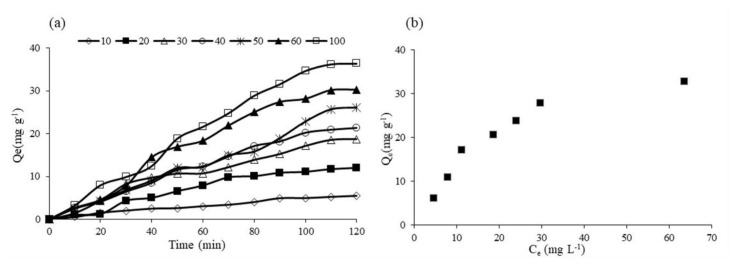
RB5 adsorption on 0.1Fe-TiO_2-_300 as (**a**) a function of time; (**b**) a function of dye concentration at equilibrium (C_e_).

Photocatalysis is an advanced oxidation process and a surface phenomenon, which leads to the decomposition of organic pollutants to CO_2_ and H_2_O. Adsorption of organic compound on to the surface of photocatalysts is a crucial step in measuring the effectiveness of photocatalysts in RB5 decolorization. 0.1Fe-TiO_2_-300 was selected, based on screening studies of iron loading and calcination temperature, to verify the adsorption mechanism. Figure 9a shows the effect of initial RB5 concentration (10 to 100 mg L^−1^) onto adsorption capacity at equilibrium (Qe) as a function of time. Qe was calculated from the experimental data of initial concentration (Ci) minus concentration at equilibrium (Ce) multiplied by volume divided by mass of the photocatalysts. Equilibrium time was determined when most of the adsorption sites are occupied and no further adsorption-desorption took place at the surface of 0.1Fe-TiO_2_-300. Similar results were reported in previous studies for the effect of initial dye concentration on the adsorption behavior of the TiO_2_ photocatalysts, where the higher percent decolorization was observed at lower concentration and with the passage of time it gets slow and becomes constant at equilibrium time [61,62]. Figure 9b illustrates the amount of RB5 adsorbed at equilibrium on 0.1Fe-TiO_2_-300 as a function of concentration at equilibrium. 

**Figure 10:**
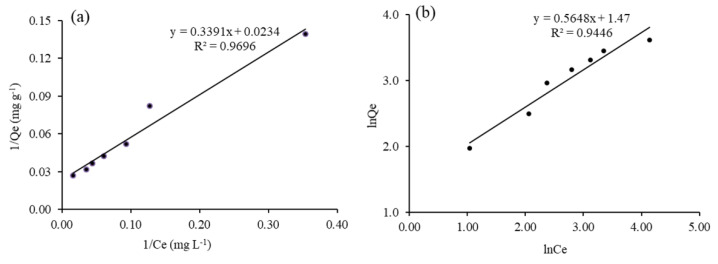
Transformation of (**a**) Langmuir; (**b**) Freundlich adsorption isotherm of RB5 decolorization using 0.1Fe-TiO_2_-300.

The distribution of dye molecules between liquid and solid phase at equilibrium can be modelled through fitting the data to different isotherm models. Our data best fitted into the linear form of the Langmuir model (plot 1/Qe vs 1/Ce), graphically described in Figure 10a. Qm and K_ads_ for 0.1Fe-TiO_2_-300 are calculated as 42 mg g^−1^ and 0.0079 L mg ^−1^ respectively. To get more overview of the surface of the synthesized material, the adsorption data was fitted into Freundlich adsorption isotherm model. Plot of lnQe vs lnCe was constructed and shown in Figure 10b. The K_F_ and 1/n were obtained as 1.78 L g^−1^ and 29.51 mg g^−1^ respectively. Summary of the isotherm constants are presented in Table 2.

### 3.8. Heterogenous Photocatalytic Kinetic Studies for Iron Doped TiO_2_ Photocatalysts

**Figure 11:**
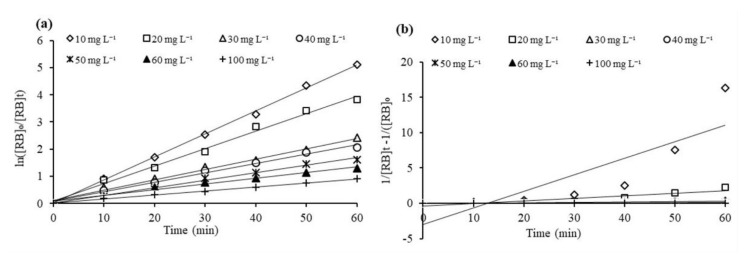
(**a**) PFO; (**b**) PSO kinetics of RB5 azo dye photodecolorization using 0.1Fe-TiO_2_-300.

Kinetic studies were performed for the photocatalytic decolorization of RB5 azo dye using Fe-TiO_2_ photocatalysts. The optimized experimental conditions such as working pH (6.2), room temperature (23 ± 1 °C), photocatalysts dose of 1 mg mL^−1^ were used for the kinetic study. For quantitative evaluation of different kinetic models, the data was plotted in pseudo-first-order (PFO) and pseudo-second-order (PSO) kinetic models. The plot of these kinetic models are given in Figure 11a,b respectively. To fit the data into PFO kinetics, the natural logarithm of the ratio ln([RB5]_e_/[RB5]) versus the illumination time (min) was fitted. The least square regression was used to calculate the K_app_ and R^2^ for each concentration, the slope of linear regression shows the apparent PFO rate constant k_app_. The kinetic data best fit the PFO kinetic model with R^2^ values ranging from 0.980–0.996 for different concentration, while for PSO fitting the R^2^ value ranges from 0.650–0.981. Therefore, the visible light driven decolorization of RB5 by 0.1Fe-TiO_2_-300 corresponds to the PFO reaction kinetics. Generally, this model is appropriate for the whole range of RB5 from few ppm (10 mg L^−1^) to higher concentration (100 mg L^−1^), in agreement with several other previous studies on the decolorization of aqueous pollutants through TiO_2_ based photocatalysts [59,60,63,64,65]. The PSO kinetics (Figure 13b) does not represent the fitting of decolorization data.

**Figure 12:**
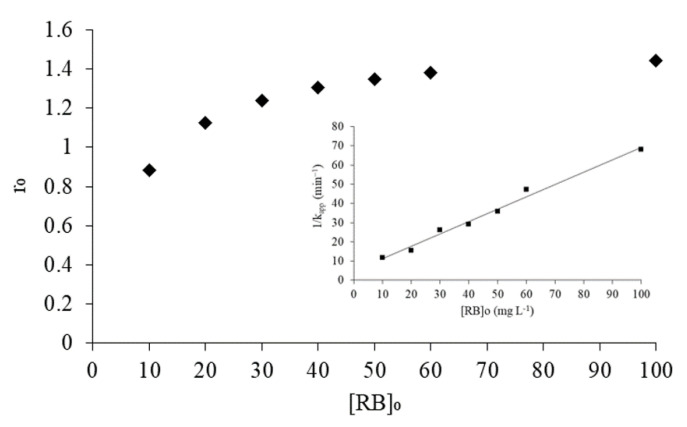
Effect of RB5 concentration on the initial rate of decolorization: Inset-Plot of reciprocal of apparent rate (K_app_).

Langmuir-Hinshelwood isotherm model (L-H) is most used to express the heterogenous photocatalytic process [66,67]. The L-H kinetic expression is shown in Figure 11. The L-H isotherm is expressed by plotting ln([RB]₀/[RB]t) verses irradiation time. The PFO constant, k_app_ (min^−1^), was calculated from the slope of the plots. To calculate the values of PFO rate constant, K_C_ (mg L^−1^ h^−1^), and L-H constant, K_LH_ (L mg^−1^), for Langmuir-Hinshelwood isotherm, the plot of 1/K_app_ against [RB5]_o_ is constructed, shown in inset Figure 12, depicted a straight line. The K_C_ value, 1.554 mg∙L^–1^∙h^–1^, obtained from the slope (1/K_C_) of the straight line (R^2^ = 0.9985) and K_LH_ 0.1317 L∙mg^–1^, obtained from intercept (1/K_C_ K_LH_), elaborate the effect of initial concentration of RB5 on the equilibrium constant for the adsorption-desorption processes. 

Figure 12 confirms that the decolorization rate of 0.1Fe-TiO_2_ augments with rising concentration of RB5, which corresponds to Langmuir-Hinshelwood adsorption model [68,69,70]. This plot showed that adsorption was the start of the photocatalytic process and clearly confirmed the Langmuir-Hinshelwood relationship.

According to the mechanism of the Langmuir-Hinshelwood kinetic model, the adsorption of the dye is a significant step in deciding photocatalytic degradation rates. The amount of RB5 molecules adsorbed on the surface of the photocatalyst are more vulnerable to decolorization during the photocatalytic phase. As reported previously, the heterogenous photocatalytic process is surface phenomenon and the radicals generated by excitation of photocatalysts could readily react with adsorbed dye molecules on the surface, moreover, this can reduce the recombination of electrons and holes and increase the photocatalytic efficiency [35,71].

### 3.9. TOC Analysis

**Figure 13:**
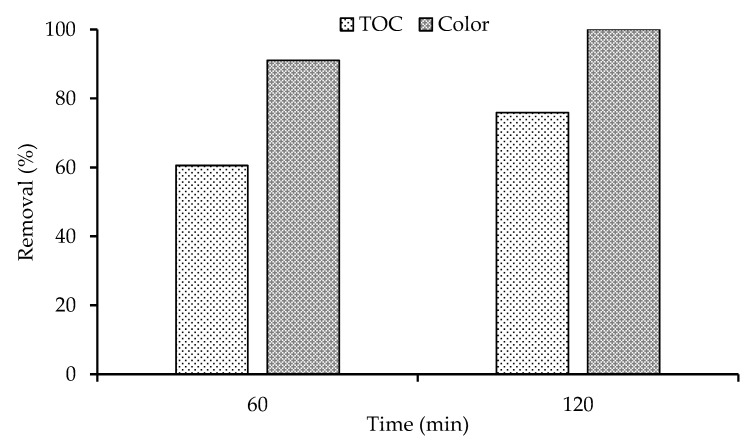
Comparison between decolorization and TOC removal of RB5 using 0.1Fe-TiO_2_-300.

TOC analysis was conducted for the optimized photocatalyst under optimized conditions including working pH, 1 mg mL^−1^ dose and 30 mg L^−1^ dye concentration at room temperature (23 ± 1). As can be seen in Figure 13. 60% TOC and 91% color removal was achieved in first 60 min for 0.1Fe-TiO_2_-300 under visible light irradiation. Prolong exposure of 120 min under visible light can eliminate the color as well as TOC, so, under the optimized conditions 0.1Fe-TiO_2_-300 photocatalysts has the ability to eliminate the RB5 dye. A similar kind of results are reported previously, 52% TOC removal was achieved for 5%Fe-TiO_2_ photocatalysts in 120 min of irradiation [58].

### 3.10. Photocatalytic Disinfection Performance Evaluation

**Figure 14:**
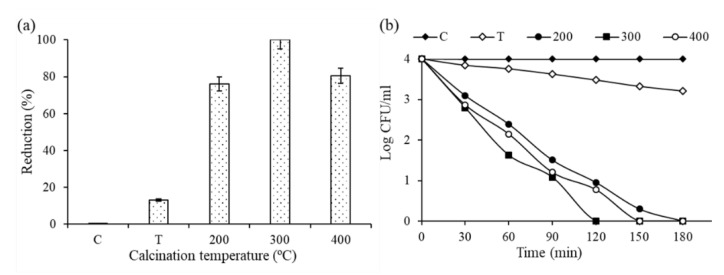
(**a**) Antibacterial performance of TiO_2_ (T) and 0.1Fe-TiO_2_ photocatalysts calcined at different calcination temperatures; (**b**) kill time analysis log CFU mL^−1^ of E. coli. C = control, T = TiO_2_-300, 200 = 0.1Fe-TiO_2_-200, 300 = 0.1Fe-TiO_2_-300 and 400 = 0.1Fe-TiO_2_-400.

Photocatalytic inhibition was conducted for 0.1Fe-TiO_2_ photocatalysts to check the antibacterial activity against selected bacterial strain *E. coli* (ATCC-25922). Experiments were conducted for control (C), bare TiO_2_-300 (T), 0.1Fe-TiO_2_-200 (200), 0.1Fe-TiO_2_-300 (300), and 0.1Fe-TiO_2_-400 (400). Control experiments are without addition of photocatalysts, and the effect of only light was observed on the deactivation of the selected bacterial strain. Initial screening results in Figure 14a shows the evidence of biocidal performance of 0.1Fe-TiO_2_ calcined at different calcination temperatures against tested bacterial pathogen. 120 min was estimated best for 0.1Fe-TiO_2_-300 during kill time analysis with maximum growth inhibition through Kill-time analysis. A noticeable viability of the test pathogens with the passage of time can be seen in Figure 14b.

### 3.11. Energy Efficiency Analysis

The energy consumption and price estimation for the removal of RB5 dye in 1000 L of wastewater was conducted through the equation 7. The energy consumption for Bare TiO_2_ was found higher as compare to 0.1Fe-TiO_2_-300. The energy consumption for iron doped TiO_2_ was 207 KWh m^−3^ while the cost was 5309 PKR (33 USD) for 1000 L of textile wastewater.

## 4. Conclusions

An efficient photocatalytic system for decolorization of double azo RB5 dye and water disinfection of *E. coli* was successfully developed. Screening studies were conducted with a series of Fe-TiO_2_ photocatalysts synthesis via sol–gel technique and characterized using TGA, FTIR, XRD, SEM coupled with EDX, TEM, DRS and BET analyses. Iron loading and calcination temperature highly affected the photocatalytic performance of the synthesized photocatalysts. Complete decolorization of RB5 azo dye was achieved by the best selected photocatalyst 0.1Fe-TiO_2_-300 at ambient temperature, solution working pH 6.2 and 1 mg g^−1^ dose in 60 min of visible light irradiation. The isotherm studies for the adsorption showed that the modified material followed the Langmuir with Q_m,_ 42.7 mg g^−1^ and K_ads_ 0.0079 L mg^−1^. Under illumination the modified photocatalytic material had higher decolorization efficiency as compared to unmodified photocatalyst. Kinetic studies of the modified material under visible light irradiation showed that the reaction follows the PFO kinetics. The illumination reaction followed the Langmuir-Hinshelwood model as the rate of dye decolorization increased with increment in initial dye concentration. The L-H constant k_c_ was 1.5542 mg L^–1^∙h^–1^ while K_ads_ was found 0.1317 L mg^–1^. Furthermore, maximum growth inhibition and photocatalytic disinfection activity of 0.1Fe-TiO_2_-300 photocatalyst showed a drastic decrease in viability of the test pathogens. Moreover, 0.1Fe-TiO_2_-300 photocatalysts was more energy efficient as compared to bare TiO_2_. It is recommended to use solar light for higher energy efficiency.

## Figures and Tables

**Table 1:** Comparative studies with Fe doped TiO_2._

Synthesis Method	Fe Contents	Calcination Temperature (°C)	Pollutant	Photocatalytic Efficiency and Reaction Time	Reference
Sol-gel	5 wt%	500	Methylene blue	55.45% 240 min	[24]
Sol-gel-Hydrothermal	0.40 wt% Fe-TiO_2_	200	active yellow XRG	88.8% (UV), 64.1% (Vis) 60 min	[15]
Sol-gel	3.0 mol%	400	*E. coli*	100% inactivation 120 min	[25]
Sol-gel	3.0 mol%	500	Rhodamine 6G	100% 40 min	[26]
Sol-gel	1.0Fe (at%)	550	methyl orange s	>80% 120 min	[27]
Sol-gel	0.15 mol%	500	RB5	100 60 min	[14]
Hydrothermal	Fe:Ti, 1:3	N.C	RB5	90% 120 min	[28]
Sol-gel	1.62%	450	RB5	100% 60 min	[29]
Co-precipitation		500	RB5	91% 60 min	[20]

**Table 2:** Summary of isotherms constants.

Isotherm Model	Plot	Parameters	R^2^
Langmuir	1/Qe vs 1/Ce	Q_m_ = 42 mg g^−1^	0.9696
		K_ads_ = 0.0079 L mg^−1^	
Freundlich	lnQe vs lnCe	*n* = 1.78	0.9446
		K_F_ = 29.51 mg g^−1^	

## Supplementary Materials

The following are available online at https://www.mdpi.com/2079-4991/11/2/436/s1, Figure S1. Light spectrum of the visible light and Figure S2. Bandgap estimation from DRS spectra.

## Appendix A. Effect of Calcination Temperature and Iron Loading

A range of Fe-TiO_2_ photocatalysts were synthesized to screen out the best combination of metal loading and calcination temperature. Different metal loading in the range of 0.01–5wt% while calcination temperatures for the activation of Fe-TiO_2_ photocatalysts were selected based on the TGA thermograms. Therefore lower (200 °C), moderate (300 °C), and higher (400 °C) temperatures were selected to analyze the effect of calcination. List of photocatalyst synthesized along with performance using iron as a dopant with different metal loading and calcination temperature is shown in following Table A1.

**Table A1:** Effect of calcination temperatures on RB5 decolorization for different Fe-TiO_2_ loadings.

Photocatalyst	Calcination Temperature (°C)		
	200 °C	300 °C	400 °C
0.0Fe-TiO_2_	40.16	47.73	28.35
0.01Fe-TiO_2_	74.46	76.11	70.37
0.05Fe-TiO_2_	78.64	82.31	79.45
0.1Fe-TiO_2_	86.63	91.06	88.63
0.5Fe-TiO_2_	79.13	86.80	84.01
1.0Fe-TiO_2_	69.53	83.39	80.72
5.0Fe-TiO_2_	61.30	79.30	75.97

Reaction Conditions: Reaction temperature 22 °C, photocatalyst dose 1 g L^−1^, pH 6.2, dye conc. 30 mg L^−1^, illumination 500 w halogen lamp.

Table A1 also explains the effect of iron loading on the decolorization capability of synthesized photocatalysts for RB5 dye. Similar trend was found for iron loading at different calcination temperatures, percent decolorization increased with increasing iron loading but it started decreasing after an optimum loading level. In our case the optimum iron loading was observed to be 0.1 weight percent, where maximum decolorization was found to be 86, 91 and 88 percent at 200, 300 and 400 °C calcination temperatures, respectively. Similar results were reported previously, where 100% RB5 decolorization was obtained for 1.65Fe-TiO_2_-450 [29]. When the iron load is high on the photocatalyst, metal oxides are formed and function as charge recombination centres which decrease the photocatalytic activity by the interference with mass transfer, avoiding the charge carrier species to continue the photocatalytic process [70,72]. Another study found that iron spread across the surface of TiO_2_ which serve as charged species trappers which decrease electron/hole pairs’ recombination rate, favouring the formation of a growing number of radical species and improving photocatalytic efficiency [68]. It is also well understood from previous studies that iron loading restrains the increase in grain size and refine the crystallite size, which ultimately increase the photocatalyst performance [73]. It can be concluded that the proposed nanoparticle i.e., 0.1Fe-TiO_2_-300 showed better decolorization performance in 60 min, low calcination temperature and low level of dopant when compared with the reported literature.
